# Do We Need a Civil Service Reform in Medicine?

**Published:** 1886-07

**Authors:** 


### DO WE NEED A CIVIL SERVICE REFORM IN MEDICINE?

No one has said so, but there has evidently been, at the Jefferson Medical College of Philadelphia, an application of political methods unworthy of so high a scientific body as that which constitutes the faculty. There have been "offensive partisans," and their want of loyalty has cost a number of subalterns their official heads. Mind, no one else has said so, and we have no *authority* for saying so; we arrive at the conclusion from indications and from disconnected facts—trivial in themselves but which put together furnish evidence that loyalty to the South and West, to the National Medical Association, to justice and to the code of our fathers has been construed into a political crime, by those in authority, and that "the punishment has been made to fit the crime," at Jefferson Medical College.

Smarting under the defeat of the scheme to organize the International Medical Congress out of the select material of Philadelphia, New York and Boston, in violation of every principle of right and justice, certain members of that august body, the faculty of the Jefferson Medical College, have doubtless cleared out of their positions, all the lecturers and assistants and adjuncts who took part in the controversy, and against the Agnew-Roberts-Hayes faction in the International Congress business; beginning with Shoemaker. We believe it was not intended to part with Pancoast, and his resignation, (which does him honor), was a surprise to the bosses. He is said to have withdrawn in disgust; that he, being democratic, and seeing his friends badly treated, could not, consistently with his self respect, longer affiliate with the *soi disant aristocrats* of the faculty—whom one of our exchanges so irreverently calls "*dudes.*"

Why do we say so? Because—first, it is known that a large number of assistant professors and lecturers have been dispensed with at the Jefferson Medical College without thanks, and as far as we know they are all of the Shoemaker-Atkinson politics—those two excellent gentlemen being first amongst them to go; 2nd. Because Pancoast did resign, and was received with open arms by those dismissed. They gave him a dinner, at which, according to the MEDICAL RECORD, none of the Jefferson Professors were present; and "so few of the prominent [?] Philadelphia Physicians" were invited.

3rd. Because those mentioned have pooled their interests—and, combining on the Medico-Chirurgical College, have already formed a new faculty, and propose to run opposition to the "Old Jeff."

Should our surmises be true we will back the new concern to the extent of our humble ability; and will urge the profession of the South and West to stand by them, as they stood by us, and suffered for opinions sake.

Verily, it is a strange inconsistency to declare so vehemently against politics in medicine, and then to be the first to apply political methods in Medical Colleges. Only a few days ago, the Apostle of the Godly self-elect electrified his ninety-nine fellow-"American Physicians," and brought about a tremendous clapping of hands, so to speak, by the declaration that "we want a society with no politics and no code,"—and behold!—

In dismissing these Southern (and Western) sympathizers the Jefferson Medical College has lost its last hold on the patronage of the South and West; and as a matter of business, this step was very unwise; it was biting the nose off to spite the face.

